# Psychiatric services for adolescents with complex mental health needs: A qualitative study of service user preferences

**DOI:** 10.1177/13591045251329151

**Published:** 2025-04-15

**Authors:** Ingrid Byng Strøm, Annika Lexén, Marianne Bonnert, Ulf Jonsson

**Affiliations:** 1 Center of Neurodevelopmental Disorders (KIND), Centre for Psychiatry Research, Department of Women’s and Children’s Health, 27106 Karolinska Institutet & Stockholm Health Care Services, Stockholm, Sweden; 2 Child and Adolescent Psychiatry, Stockholm Health Care Services, Stockholm, Sweden; 3 Department of Health Sciences, 5193 Lund University, Lund, Sweden; 4 Centre for Psychiatry Research, Department of Clinical Neuroscience, 27106 Karolinska Institutet & Stockholm Health Care Services, Stockholm, Sweden; 5 Department of Medical Sciences, Child and Adolescent Psychiatry, Uppsala University, Uppsala, Sweden

**Keywords:** Adolescents, CAMHS, service user involvement, FACT, implementation, participation, qualitative study

## Abstract

**Background:**

Child and Adolescent Mental Health Services (CAMHS) currently seeks to implement service models that are better suited for young service users with complex mental health needs. This study explored what adolescents view as the key components of a well-functioning contact with CAMHS.

**Methods:**

This study was conducted as part of the ongoing implementation of Flexible Assertive Community Treatment (FACT) in the CAMHS in Stockholm, Sweden. Interviews were conducted with 14 adolescents (64% females) aged 14 to 18 (*M* = 15.78, *SD* = 1.32) with intensive or longstanding contact with CAMHS. The interviews were analyzed using reflexive thematic analysis.

**Results:**

Five key themes were generated: (1) “being involved in my care” – receiving relevant information and being part of important care decisions; (2) “accessing care when I need it” – ensuring services are easily accessible and present in the community; (3) “building a good relationship” – developing strong connections with mental health professionals; (4) “sharing the burden” – improving support and organization to reduce stress related to the healthcare system; and (5) “personalizing my care” – tailoring care to individual preferences and circumstances.

**Conclusions:**

The findings emphasize the importance of engaging young service users in developing a mental health service that meets complex needs.

## Plain Language Summary

Many young people have mental health conditions that cause problems in several parts of their daily lives. This study explored what they find important in their contact with mental health services. We interviewed 14 adolescents in Sweden with lived experience of mental health care. The adolescents wished to be informed about their care and engaged in important decisions. They also found it important that the services were available when they needed them. Some commented that child and adolescent mental health services should be more present in the community. A good relationship with the mental health professionals was highly valued. Many described that it could be difficult to understand and handle all their different service contacts and that they would like a more user-friendly service system. Finally, the adolescents wanted the care to match their personal needs. We conclude that this type of input from young people is necessary to help build a child and adolescent mental health service for the future.

## Background

A significant group of children and adolescents experience complex mental health needs, with persistent challenges that affect their functioning across several life domains and require a wide range of services and support (Georgiades et al., 2019; Winiarski et al., 2020). This underscores the importance of carefully planned actions to coordinate services, shorten hospital stays, and reduce the use of coercive measures. Currently, mental health services strive to implement service models that are more finely tuned to the specific needs of young service users (Svensson et al., 2017). However, the field faces several challenges, including a shortage of child and adolescent psychiatrists and diminishing resources in CAMHS across many countries (Dubicka & Bullock, 2017; World Health Organization, 2021). In the past few years, Sweden has seen a notable increase in adolescents seeking care from Child and Adolescent Mental Health Services (CAMHS; National Board of Health and Welfare, 2021). Notably, the number of girls receiving inpatient care has risen by 19%, and the number of children receiving care under the Compulsory Psychiatric Care Act has increased by 46% since 2017 (National Board of Health and Welfare, 2022). Similar trends have been observed in countries worldwide, indicating a global youth mental health crisis (McGorry et al., 2024).

In view of these challenges, experts advocate a holistic multisector mental health policy in which resources are expended wisely and where evidence-based practices lay the foundation (Skokauskas et al., 2019). Such an approach would focus on comprehensive services across society, including diagnostic assessments and treatment, prevention, early intervention, and rehabilitation services (Skokauskas et al., 2019). A recent review of service models for adolescents and young adults with severe mental illness further underscores the complexity of service delivery for this target group (Woody et al., 2019). The key components identified included multidisciplinary teams, collaborative care, multiagency collaboration, services addressing comorbidities, and early discharge planning. No single type of service was considered sufficient to provide all the healthcare and support needed, and the authors emphasize the importance of considering developmental stages and fluctuations in the severity of the illness.

When new service models are developed and implemented, involving service users throughout the process is crucial. Children and adolescents have the right to express their preferences and actively participate in decisions relevant to their care (World Health Organization, 2012). It has also been reported that service users’ sense of agency and participation significantly impact their ability to recover from mental illness (Bjonness et al., 2020; Slade, 2017). Service user involvement in mental health care is gradually becoming integral to clinical research (Ali et al., 2023; Mawn et al., 2015; Meldahl et al., 2022). A recent systematic review on user involvement in adolescent mental health care (Viksveen et al., 2022) found that adolescents who were not given the opportunity to participate felt unheard and perceived that their treatment was not tailored to their individual needs. In contrast, those engaged in collaborative therapeutic relationships experienced open communication and shared decision-making. This approach fostered trust, increased their engagement in their own care, and led to reduced dropout rates. The review also explored involvement at an organizational level, highlighting the outcomes relevant to service users and assessing whether the conditions for involvement were optimal (Viksveen et al., 2022).

The present study was conducted as part of the preparatory work for implementing Flexible Assertive Community Treatment (FACT) for adolescents with complex mental health needs that require extensive and coordinated care in CAMHS in Stockholm, Sweden. FACT is an adaptation of Assertive Community Treatment (ACT) that was originally developed for the most severely affected 20% of individuals with severe mental illness at risk of hospitalization. In contrast to ACT, FACT targets a broader population, providing the most intensive care to those with the most severe needs through a team-based approach (van Veldhuizen & Bähler, 2013). FACT is a recovery-oriented integrated care and support model in which participation is a key concept and care is adapted to service users’ needs (van Veldhuizen & Bähler, 2013). The service model has previously been implemented for adolescents in the Netherlands (van Veldhuizen, 2007) and Norway, where young service users prefer contact with a youth FACT team to usual healthcare services (Nord-Baade et al., 2022).

To guide this implementation, adolescent service users’ perspectives will be crucial. Several previous studies have explored adolescents’ experiences of mental health care, providing valuable insights. Relational aspects (e.g., being heard, respected, and experiencing continuity), the content of the care (e.g., youth-friendliness, facilitating communication, receiving relevant advice, and developing self-care skills) and being involved are recurring themes in the literature (e.g., Burnand et al., 2024; Buston, 2002; Harper et al., 2014; Hartzell et al., 2009; McCann & Lubman, 2012; Meldahl et al., 2022; Nord-Baade et al., 2022; Persson et al., 2017; Viksveen et al., 2022). Parental involvement is also a central theme in some studies (Harper et al., 2014; Hartzell et al., 2009), along with accessibility and personalization/tailoring of services (e.g., Kirk et al., 2023).

Similarly, the few prior studies on experiences from the CAMHS in Sweden highlighted the importance of accessibility, the usefulness of the session, and being heard, seen, and accepted (Hartzell et al., 2009; Persson et al., 2017). This has also been confirmed by a survey conducted by a Swedish organization for neurodevelopmental conditions, indicating an association between experiencing care meetings as being helpful and feeling engaged in the care process. However, only 28% expressed satisfaction with the information they received, and nearly half felt they were not fully heard (Swedish National Association Attention, 2016).

Building on this existing body of research, we sought to understand how those with the most complex needs currently perceive their encounter with CAMHS and explore their thoughts on improving their care.

## Methods

### Design

This qualitative study was based on semi-structured interviews with 14 adolescents with ongoing contact at CAMHS in Stockholm, Sweden. The interviews were analyzed using reflexive thematic analysis (Braun & Clarke, 2022). This inductive approach, using open and organic coding, was chosen to reflect the diverse experiences of adolescents. It also highlights the researchers' active role and their clinical expertise in interpreting the interviews and generating themes (Braun & Clarke, 2020). The study was reported in accordance with the Consolidated Criteria for Reporting Qualitative Research (COREQ), a 32-item checklist for interviews and focus groups (Tong et al., 2007).

### Study setting

FACT is currently being implemented in 4 units within the Section for Intensive Outpatient Care at CAMHS, Stockholm. This specialty care section focuses on children and adolescents with complex mental health needs and significant functional impairments who require extensive and coordinated care. Each unit consists of multiprofessional teams providing various services adapted to adolescents’ needs.

### Recruitment and participants

The goal was to recruit a diverse sample of 10–20 adolescents, which was considered feasible and sufficient to provide a variety of experiences. Adolescents received information about the study through their contact person at CAMHS. The contact persons were informed of the study by their unit manager and encouraged to inform all eligible adolescents about it. Interested service users received written information about the study and contact information. Written consent was collected by their CAMHS contact person and forwarded to the research team. Subsequently, the research team contacted the adolescents and scheduled an interview. Eligible adolescents were aged 12-18 and had ongoing treatment either at the Section for Intensive Outpatient Care or a longstanding contact with regular outpatient care. Adolescents who had initiated contact with CAMHS but had not yet received any form of treatment were not eligible to participate.

### Data collection

A semi-structured interview guide used when implementing FACT in Norway (Høiseth et al., 2020) was adapted to fit the Swedish healthcare context and service users with complex mental health needs and a high degree of disability. Adaptations were made by reducing the number of questions, adding closed-ended follow-up questions, and providing examples if necessary. This enabled more diversity in the sample, allowing less verbally expressive adolescents to participate. Sociodemographic characteristics were gathered at the beginning of the interview. The interview guide consisted of open and narrowed-down questions about how the adolescents experienced the existing service system. The questions dealt with a broad range of topics relevant to the adolescents’ experiences of the service system. The topics included their impression of the existing service system and thoughts about improvements to the system, what defines a good mental health professional, their experiences of safety, and the possibility of involvement (see Supplemental Material). To ensure credibility, the adolescents were asked follow-up questions and were encouraged to provide examples (Table 1).

**Table 1.** Examples of Questions From the Semi-structured interview Guide.

Question	Follow-up	Examples (when necessary)
What is important for your well-being?	How can CAMHS help with that?	Assistance in leisure activities, school, family, friends, health, body, identityetc.
What does it take to feel safe with the healthcare staff you meet at CAMHS?	What should they do? What do you need to know about them?	Prepare sessions, things to say, read your medical journaletc.
What defines a good mental health professional?	Reflect on whether you have previously met a mental health professional who was good at helping you. What did that person do that was effective?	Choose personal examples from previous statements during the interview
Which parties in your network need to cooperate/communicate more so that you feel better?	Why is it important that they talk? How should they cooperate?	Parents, teachers, siblings, other relatives, coaches, case managersetc.

*Note.* CAMHS: Child and Adolescent Mental Health Services.

Data was collected from December 2022 to April 2023. Interviews were conducted at the adolescents’ local clinic, except for two interviews held digitally upon the adolescents’ request. All the interviews were held individually and lasted approximately 30–45 minutes. The interviews were digitally recorded with the adolescents' consent. The data were stored in a secure file area.

### Data analysis

The interviews were performed by a Clinical Psychologist (IBS) employed by CAMHS. The Principal Investigator (UJ), a Clinical Psychologist and researcher, was also employed by CAMHS. A Clinical Psychologist and researcher (MB) and an Occupational Therapist and researcher (AL), both external to CAMHS, were involved in interpreting the results. Consequently, the analysis represents interpretations from clinicians’ perspectives. All researchers had prior experience in using qualitative methods.

The interviews were transcribed verbatim by 4 transcribers, who all received instructions to produce a complete, written version of the spoken words. The data were analyzed using Reflexive Thematic Analysis (RTA) as described by Braun and Clarke (2022) and exemplified by Byrne (2022). The process included the following six steps: (1) getting to know the data, (2) initial coding, (3) generating themes, (4) reviewing themes, (5) defining and naming themes, and (6) compiling the results. First, two authors (IBS and MB) listened to the audio recordings, took preliminary notes, and read the transcriptions. Second, the initial coding of all the relevant data for the aim of the study was performed by one researcher (IBS), and the preliminary codes were further developed, reviewed, and compared with those of the other authors. Third, patterns in coding and meaningful units were identified across the dataset, and themes were generated from the codes. Fourth, the themes were reviewed, and the different qualities of the generated themes were assessed. During steps three and four, the authors had several meetings to discuss topics related to coding, patterns in the dataset, and possible themes. The coding approach was collaborative and reflexive, designed to develop a richer and more nuanced reading of the data. Fifth, a final comparison of the themes was performed against both the dataset and the study aim, and the theme names were revised. Pertinent quotations were used to exemplify the emerging themes. One author (IBS) kept a diary throughout the process to increase reflexivity (Korstjens & Moser, 2018).

## Results

### Study population

Interviews were conducted with 14 adolescents (64% females) aged 14 to 18 (*M* = 15.78, *SD* = 1.32). The adolescents had a range of mental health conditions and often multiple ongoing contacts; several had undergone inpatient treatment (Table 2). During the interviews, the majority described complex psychosocial circumstances such as school absence, problems with peers, and contact with social services.

**Table 2.** Sociodemographic Characteristics of the Participants (*N* = 14).

Characteristics	*N* (%)
Age	
14	3 (21)
15	3 (21)
16	4 (29)
17	2 (14)
18	2 (14)
Gender	
Female	9 (64)
Male	5 (36)
Other	0 (0)
Reason for CAMHS contact	
One diagnosis reported	5 (36)
Two or more diagnoses reported	9 (64)
Depression	7 (50)
Anxiety (including GAD, panic syndrome, agoraphobia, social phobia)	9 (64)
OCD	2 (14)
PTSD	3 (21)
Autism	5 (36)
ADHD/ADD	4 (29)
Other (including eating disorder, borderline personality disorder, psychosis, and self-harm)	4 (29)
Type of unit^a^	
Outpatient unit	11 (79)
Intensive outpatient unit^b^	13 (93)
Other^c^	7 (50)

*Note.* ADD: Attention Deficit Disorder; ADHD: Attention Deficit Hyperactivity Disorder; CAMHS: Child and Adolescent Mental Health Services; GAD: Generalized Anxiety Disorder; OCD: Obsessive Compulsive Disorder; PTSD: Post-Traumatic Stress Disorder.

^a^Some participants had contact with more than one unit.

^b^Units specifically targeting adolescents with complex mental health needs.

^c^Including inpatient units, psychiatric emergency units, and units specializing in eating disorders.

### Themes

Five main themes were generated: (1) being involved in my care; (2) accessing care when I need it; (3) building a good relationship; (4) sharing the burden; and (5) personalizing my care. Below is a description of the themes and subthemes, with pertinent quotations. The themes are presented in the order in which they appeared in the analysis. Examples of the four levels of analysis (theme, subtheme, code, and meaningful unit) are presented in Table 3.

**Table 3.** Examples of the four Levels of Analysis.

Theme	Subtheme	Code	Meaningful unit
Being involved in my care	Being involved in treatment decisions	Lack of involvement creates insecurity	“I think that if things are predetermined and have not truly been discussed before in a more open way so that you get to participate a bit more, then it becomes like this, you feel a little bit put off.” Participant 2
Accessing care when I need it	Experiencing continuity and accessibility	Request for crisis counseling after office hours	“I think many people have anxiety in the evenings… and when they are going to sleep. That you can chat a little with (staff) and tell them how you feel and maybe get a little (help) with what you can do to make it better. Or what you can do to be able to fall asleep.” Participant 12
Building a good relationship	A professional commitment to me	Being treated as more than a mental illness	“Tell us a little about yourselves. Ask about the young person’s life outside of illness and diagnosis. Get to know the youth.” Participant 6
Sharing the burden	Coordinated services and close communication	Burdensome to repeat one’s story	“It can be very difficult to hear yourself say those things.” Participant 1
Personalizing my care	Adjusting care to my current circumstances	Aligning frameworks for care contact with the individual’ preferences	“Going for a walk or meeting at the unit would be my preferred choice.” Participant 11

### Being involved in my care

All the adolescents expressed that it was important for them to be informed and consulted when significant decisions were made about their care.

#### Being involved in treatment decisions

Adolescents emphasized that they wanted to be involved in designing and formulating goals, plans, and strategies for their treatment. Many examples were given of how participation led to increased motivation.

“It feels like it is important that the person in question gets to be involved and pull the strings. That no one should sort of do it for them, not parents, not the psychologists; they should not decide things for the (adolescent).” Adolescent 12

Several adolescents had experienced a lack of involvement in their own care. They expressed that the treatment plan was often presented to them without allowing them to have a say.

“My doctor suggested it first. A drug that I had already researched and sort of read about… and the reviews. And I knew it would not work because it did not treat social anxiety, so I told (them), but they kind of told me that they know what's best. Because they're a doctor. Although I tried it and it did not work, so it only got worse.” Adolescent 1

#### To be trusted and included when information is shared

Adolescents expressed a desire to be informed about whether information needed to be shared through the adolescent’s support network or staff members involved with the adolescent. The experiences of mental health professionals who share sensitive information without the consent of their youth resulted in feelings of insecurity. Conversely, transparency regarding information sharing and the methods by which it occurs foster feelings of trust and security. Many emphasized the significance of safeguarding adolescents’ privacy.

“The journal only gives the healthcare provider's point of view of what has happened. And it could be completely wrong. And then (staff) form an opinion based on that, and you might get the wrong idea before you meet the youth. In my case, there was a lot of stuff in my journal that was maybe, it was not correct information. But the only one who could fix it was me, and I was not trusted. Which you can understand to a certain extent, but still…” Adolescent 14

### Access to care when I need it

The desire for increased accessibility was shown in a variety of ways through the adolescents’ stories.

#### Experiencing continuity and accessibility

Most adolescents expressed a desire to be able to contact their mental health professional beyond the clinic’s opening hours, either via phone calls, SMS, or chat. The adolescents described that they needed immediate support from a mental health professional when difficult thoughts and feelings arose, not during a scheduled appointment a week later.

“When you're alone in the evening, and all the emotions just kick in. (…) A whole day has gone by, and you have spent the whole day distracting yourself. (…) When a week has passed, I will have forgotten everything that happened.” Adolescent 4

Other adolescents expressed that they would like more consistent contact with their mental health professional and the possibility of reaching out to the same mental health professional during times of heightened distress. Many found it challenging to meet new mental health professionals repeatedly. Conversely, some adolescents described forming such a strong connection with staff that discontinuing their CAMHS involvement proved challenging.

Several adolescents opined that CAMHS has a poor reputation among young people. Many shared their experiences of having to work hard to access CAMHS, and during the waiting period, they experienced a decline in both their mental health and their daily functioning.

“For me anyway, it was very difficult to get in contact with CAMHS when I truly needed to. (…) I know that they have very long waitlists and it’s kind of hard to get around, but... I would probably still say that if they identified more young people at an earlier stage, youths could get help in time so that it does not have to (lead to) inpatient care or so.” Adolescent 10

#### Proactive services in society

The majority of adolescents emphasized that CAMHS could play a more active and preventive role in society. They often felt that CAMHS was missing in the arenas where adolescents live. Many adolescents expressed that they would like to see CAMHS establish an active presence on social media platforms, such as maintaining an account with regular updates or running advertisements. One youth stressed the importance of feeling that CAMHS is there for you when needed.

“Tell youths how to get help if you feel bad, how to get help if you have an eating disorder, how to get help if you have anxiety, panic disorder, how to get help if you have perhaps been sexually abused, or the like. Show us that CAMHS, we are here for you, we want to listen.” Adolescent 12

Conversely, some adolescents wished to keep their contact with CAMHS separate from their social life. Importantly, adolescents requested that CAMHS take the initiative to destigmatize mental health conditions by sharing their knowledge with young people and adults in their surroundings, including school staff.

### Building a good relationship

All the adolescents stressed the importance of their relationship with the staff. Some even described the relationship as more important than the treatment itself.

#### A professional commitment to me

Adolescents conveyed that they wanted a personalized connection with their mental health professionals, emphasizing the importance of reciprocity in the therapeutic relationship (e.g., staff showing genuine interest in the adolescents as individuals, not merely as service users). Many adolescents characterized their relationship with mental health professionals as extending beyond a formal professional encounter. Some also articulated that the staff's personal engagement gave them a sense of security.

“Staff should not only talk about what is difficult; you should be... curious in a way. How are you? Did you do something fun, a bit more like that. (…) Personally, I would feel more comfortable. We have a slightly more friendly relationship. (…) (Mental health professionals) shouldn’t be just any dentist you meet; they should be someone... one can feel comfortable with. Almost like an extra sibling, so to speak.” Adolescent 3

Several adolescents shared their experiences of being treated improperly by mental health professionals, including feelings of disrespect, a lack of empathy, not being heard, and not being allowed to finish what they wanted to say. Such encounters had a negative impact on their motivation for treatment, their sense of security, and their overall trust in CAMHS.

“One time, (my therapist) asked me, So, what are we doing here, do you have anxiety or what did we say? Don’t ask me if I have anxiety or not when we’ve already talked about it on several occasions.” Adolescent 10

Adolescents emphasized the need to be treated with respect. They highlighted the mental health professional’s ability to intuitively adapt to each individual's unique needs and preferences, including the use of humor. Furthermore, the staff's capacity for empathy fostered greater trust, ultimately enhancing treatment adherence.

#### Staff sharing lived experience

All the adolescents expressed positive views on staff sharing lived experience (e.g., through peer support). Many found that staff members with first-hand experience of mental health conditions were better equipped to comprehend their position, thereby conveying empathy and genuine compassion. Some adolescents welcomed the idea of meeting with a peer supporter as a component of their CAMHS interactions, while others expressed that they would like their designated service contact to share personal experiences of mental health conditions as part of the treatment process.

“That (mental health professionals) have felt sorrow, that you have had some difficult feelings, that you have felt lonely. If you can see it in their eyes, you gain more respect when you sit opposite, and it feels like she or he takes it more seriously, somehow.” Adolescent 14

### Sharing the burden

According to the adolescents, being a service user was often overwhelming, and some aspects of the healthcare system were seen as additional burdens.

#### Coordinated services and close communication

Most adolescents wished for mental health professionals to collaborate with the support network around them. Many felt that it was important for their families to obtain information about their struggles. Adolescents described it as burdensome to communicate their emotions and need for support, and several believed CAMHS could alleviate this by assuming a greater share of collaborative responsibility.

“(CAMHS and my network) need to cooperate because my mood affects all aspects of my life. Therefore, what happens at home will affect how I feel at school and vice versa. I think it would be good if, from the school's side and care units' side, it is their responsibility to cooperate primarily. Because not everyone has parents who can take that kind of responsibility.” Adolescent 2

A major concern for the adolescents was how CAMHSs communicate internally when multiple mental health services were involved. Many adolescents were accustomed to repeating their stories to numerous healthcare professionals. The lack of continuity in their care often left them feeling frustrated and abandoned, sometimes resulting in worsening of their condition.

“The emergency psychiatric clinic told me that 'there is nothing we can do here, you have to go to the outpatient care and seek help there', and the outpatient care said 'well, this will take time, and if it becomes urgent, you have to go to the emergency psychiatric clinic'. I went back and forth several times, and eventually, I attempted suicide after a very intense week where I had been to the emergency clinic at least once feeling very rejected and not so well treated and I felt so dejected, and yeah, that was it.” Adolescent 2

#### Safety issues are crucial

The adolescents also addressed the need to be protected from harm while in contact with CAMHS. Some adolescents shared experiences of suboptimal care and incidents where they were at risk of encountering harm during treatment, including mental health professionals crossing boundaries and acting unprofessionally.

### Personalizing my care

All the adolescents expressed that mental health professionals must consider their individual needs.

#### Adjusting care to my living circumstances

The adolescents saw customizing care to fit into their daily lives as an important part of their recovery. For instance, they talked about how their care helped them return to daily activities, such as exercise or attending community events.

“For those feeling better, I think group activities are a good idea; you do not have to talk, just be part of a group. And if you want to share, maybe a few words. Or do not even share; just sit there. If CAMHS could have more groups like that, more social connections, that would be great.” Adolescent 7

#### Tailoring care to my specific needs

Adolescents expressed that their interactions with CAMHS ideally should be customized based on their individual needs and preferences. For instance, adolescents held varying preferences regarding the structure of their treatment plans and individual goals. Some preferred using tools such as mobile apps for breathing exercises, while others preferred receiving printed materials to take home. Several adolescents shared experiences of struggling to comprehend the content of their treatment or failing to grasp the purpose of certain exercises. Others requested reminders prior to their appointments. One adolescent underscored the importance of acknowledging individual differences, even when diagnosed with the same conditions or facing similar challenges.

“See the person for who they are, and not just 'here we have an autistic person!' Instead, here, we have a person with autism and understand that everyone functions differently, regardless. I have autism; my friend has autism; we function completely differently. Be curious about how this individual works. I think that is important.” Adolescent 11

Adolescents held varying opinions regarding the location and timing of their therapy sessions. Many emphasized that sessions should be individually tailored to each youth’s unique circumstances and preferences.

## Discussion

This qualitative study explored what adolescents with complex mental health needs find important in their contact with CAMHS. The interviewed adolescents stressed the value of being listened to and involved in care decisions. They also desired accessibility beyond visiting hours and that CAMHS should have an active presence in the community. Great importance was attached to the quality of the relationship with the mental health professional, and adolescents talked about how this relationship affected their treatment. Furthermore, many found navigating and understanding the CAMHS system burdensome and requested support. Finally, they emphasized that their care should be more fully adapted to their specific needs and preferences.

Our findings closely mirror a European study conducted two decades ago, which identified trusting and stimulating relationships as the highest priority, followed by tailored treatments and service accessibility (van Weeghel et al., 2005). The adolescents in our study emphasized the importance of trust and personal connections with mental health professionals, aligning with findings from other countries (Burnand et al., 2024; Kirk et al., 2023; Nord-Baade et al., 2022). This issue may be particularly important for individuals with complex mental health needs (Evans et al., 2024). The need for trust may partly stem from extensive interactions with the healthcare system, including negative experiences such as frequent staff changes (Coyne et al., 2015), and possibly from family conflicts and insecure attachment histories (Alaie et al., 2020; Rognli et al., 2020). Accessibility and service user involvement were also underscored by the adolescents, consistent with prior research on children and adolescents (e.g., Bjonness et al., 2020; James, 2007; Kirk et al., 2023; Meldahl et al., 2022; Slade, 2017). Furthermore, tailoring services to individual needs, circumstances, and preferences emerged as a crucial consideration, reinforcing the need for flexible and adaptable care models in CAMHS. It is important to recognize that relationships and easy access to therapists are essential elements in therapeutic approaches designed for this target group, including Dialectical Behavior Therapy (DBT), where these aspects are fundamental (Lynch et al., 2006). Recognizing and integrating these aspects into mental health services, in general, may significantly enhance the quality and effectiveness of care for adolescents with complex mental health needs.

The wish to be unburdened has been less prominent in previous studies. Still, this finding aligns well with recent official reports in Sweden, underscoring the need to strengthen cooperation between healthcare and social services for children. (Health and Social Care Inspectorate, 2021; Swedish Government Official Reports, 2021). The educational sector has also been identified as a key player in promoting mental health (Jeindl et al., 2023). Integration and coordination of services could be particularly important for adolescents with complex mental health needs receiving services from multiple stakeholders, especially since they are at increased risk of persistent school absenteeism (John et al., 2022). As a healthcare model designed to bridge the gap between services, FACT may contribute towards meeting these needs (van Veldhuizen & Bähler, 2013). Relatedly, some adolescents raised safety concerns regarding staff crossing professional boundaries and behaving unprofessionally. Although these issues may not be sufficiently studied, they resonate with findings from a previous study on mental health professionals' experiences of adverse effects related to psychological treatment in Swedish CAMHS (Jonsson et al., 2016).

### Future directions

Continued efforts to involve adolescents will be important throughout the ongoing implementation of youth FACT teams. It will also be important to address the needs and preferences expressed by the adolescents, potentially by incorporating available strategies such as peer support (de Beer et al., 2024; White et al., 2020), shared decision-making (Bomhof-Roordink et al., 2019), and case management (Dieterich et al., 2017).

Additionally, it will be essential to explore how CAMHS can increase its visibility and accessibility (Coyne et al., 2015; Meldahl et al., 2022). This includes reaching out to groups where negative attitudes toward mental health services may prevent children and adolescents from obtaining care, as highlighted by our results. Improved coordination of health and social care could also help ensure that adolescents with complex care needs who do not already have an established treatment contact can access the services they need.

### Limitations

Our results should be viewed in the light of some limitations. First, due to the relatively small number of interviewees, it is unlikely that we have captured this heterogeneous group of adolescents' full range of views and experiences. It also remains uncertain how these findings apply to other target groups (e.g., adolescents receiving treatment for mental health issues in primary care), different healthcare systems, and various cultural contexts. Experiences and preferences may also change in response to societal and cultural trends. Given this complexity, our ambition was to provide an interpretation of views from adolescents with diverse stories, challenges, ages, and difficulties while accepting the uncertainty of sample saturation in qualitative research (Braun & Clarke, 2019). However, by sharing experiences from various contexts, including low- and middle-income countries, systematic review methodology may eventually enhance our understanding of differences and similarities across settings, populations, and perspectives. Second, dependability through methodological triangulation, by, for instance, including the perspectives of caregivers and mental health professionals, was not used in this study. In addition, member-checking through feedback from the interviewees during the process may have increased the confirmability and overall trustworthiness of this study (Korstjens & Moser, 2018). Third, preliminary coding was performed by one author (IBS), emphasizing the subjective nature of interpretations (Braun & Clarke, 2020). To enable a richer interpretation of meaning, we discussed the analytic process within the research team and included a second author’s (MB) independent review of the material. Consequently, theoretical preunderstanding related to clinical roles should be expected, which may be both a limitation and a strength of the study.

## Conclusion

The results underscore the importance of engaging adolescents in the care provision process. This study and the existing body of research it builds on should be considered in the further development of a service directed toward adolescents with complex mental health needs. The results underscore the need for holistic, comprehensive mental health services to ensure that vulnerable youth receive the full range of treatment and support they need to recover and reach their full potential.

## Supplemental Material

Supplemental Material - Psychiatric services for adolescents with complex mental health needs: A qualitative study of service user preferences Supplemental Material for Psychiatric services for adolescents with complex mental health needs: A qualitative study of service user preferences by Ingrid Byng Strøm, Annika Lexén, Marianne Bonnert and Ulf Jonsson in Clinical Child Psychology and Psychiatry

## Data Availability

The datasets generated and analyzed during this study are not publicly available to protect individual privacy. The audio files and transcribed texts are retained in a secure file area*.
