# Supplementary material for: Psychiatric services for adolescents with complex mental health needs: A qualitative study of service user preferences
Source: Clin Child Psychol Psychiatry. 2025 Apr 15;30(4):832–49. doi: 10.1177/13591045251329151 (PMC12436989; doi:10.1177/13591045251329151)
Supplement: Supplemental Material - Psychiatric services for adolescents with complex mental health needs: A qualitative study of service user preferences [file sj-pdf-1-ccp-10.1177_13591045251329151.pdf]

Supplemental material: Interview guide

1) Introducing ourselves.

2) The purpose of the study:

- Child and Adolescent Mental Health Services (CAMHS) will implement a new healthcare model called Flexible Assertive Community Treatment, FACT. FACT consists of a team of health care professionals who are trained to help children and adolescents with several types of health care needs, such as medical doctors who can prescribe medicines, psychologists who can help when you have difficult thoughts and feelings, occupational therapists who can help you make everyday life easier, a person with personal experience of receiving help from CAMHS (called Peer Support), social workers who know how to cooperate with the school and so on. Their goal is to help children and adolescents who are struggling with mental health issues and who need several different types of help at the same time (e.g., with both anxiety, problems at school, problems at home, etc.). CAMHS now want to learn more about what you and other adolescents find important when you need help from CAMHS.

- All children and adolescents in contact with CAMHS have the right by law to influence and say what they think about the treatment they receive. When you tell us about your experiences and what is important to you, you help CAMHS become better at helping and understanding others with a CAMHS contact.

3) Brief review of consent form.

4) Initial questions:

- Age and gender

- Which unit are you in contact with today?

a. CAMHS Open care

b. CAMHS Intermediate care

c. The daycare unit

d. CAMHS DBT team

e. CAMHS psychosis and bipolar disorder unit

f. CAMHS Trauma unit

5) Semi-structured interview (see Table S1).

Table S1. Questions from the semi-structured interview.

Question	Follow-up	Examples (when necessary)
What are you receiving treatment for? Briefly describe your contact with CAMHS.	How satisfied are you with the contact you have had with CAMHS so far?	Can you tell us about an occasion when you thought the contact with CAMHS worked well?<br>Can you tell us about an occasion when you thought the contact with CAMHS worked less well?
Imagine that you were free to decide what help you received from CAMHS. What kind of help would you want?	What is important for your well-being? How can CAMHS help with that? Is there anything that CAMHS is not doing today that you would like them to do? If yes, then what? What do you think other young people in contact with CAMHS would be helped by?	Assistance in leisure activities, school, family, friends, health, body, identity, etc.
Which parties in your network need to cooperate/communicate more for you to feel better?	Why is it important that they talk? How should they cooperate?	Parents, teachers, siblings, other relatives, coaches, case managers etc.
What defines a good mental health professional?	Reflect on whether you have previously met a mental health professional who was good at helping you. What did that person do that was effective?	Choose personal examples from previous statements during the interview.
What does it take for you to feel safe with the healthcare staff you meet at CAMHS?	What should they do? What shouldn't they do? What do you need to know about them? What can make you feel insecure about staff?	Prepare sessions, things to say, read your medical journal before sessions. Repeating your story to several staff. Feel free to give examples of your own experiences.
What is your opinion about getting support from staff who have had their own contact with CAMHS in the past?	What could be good about it? What could be bad about it?	
If you were to decide, where would you like to meet staff from CAMHS for your sessions?		At the unit, at home, taking a walk, online meetings, other places.
How can CAMHS reach out to young people who need help?		For example, young people who are at home feeling bad, but have no contact with

		CAMHS.
What parts of your CAMHS contact do you want to be involved in and influence?		Goals, how often meetings are held, where and when to meet, how long the visits should be, what treatment you receive; medication and psychological treatment, who should participate in meetings concerning you, how to involve your parents etc.
How do you wish to express your opinions about the CAMHS contact?	Orally/by talking to mental health professionals that you're in contact with?<br>In writing? (By texting or writing down during visits)<br>Through surveys?<br>Other methods of feedback?	
When planning the treatment at the beginning of the CAMHS contact - how would you prefer it to be done?	What can be difficult about planning?<br>What kind of goals do you want to set?	Do you have any good experiences in making a plan? If yes, what did you do then? If not, what was missing?
In your opinion, who should be informed about what you tell your staff at CAMHS?	What information may be shared with all mental health professionals you meet at CAMHS?<br>When necessary, how should mental health professionals share information about you with others?	Parents, school, social services etc.
How do you prefer using digital services in contact with CAMHS?	What could be good/bad about it?	For example, chat, sleep diary apps, practice breathing exercises, social media, etc.
If you could choose freely, when would you like to be able to contact mental health professionals at CAMHS?	At what times? Why then?<br>What kind of help do you need in the evening? What could be good/bad about talking to mental health professionals in the evening?	
